# Investigating Parental Observations of Early Autism Development in Simplex and Multiplex Families

**DOI:** 10.1007/s10803-024-06262-0

**Published:** 2024-04-20

**Authors:** Dominique B. Cleary, Murray T. Maybery, Hannah Waddington, Ella Macaskill, Andrew J.O. Whitehouse

**Affiliations:** 1 Telethon Kids Institute, University of Western Australia, Subiaco, Australia; 2 School of Psychological Science, The University of Western Australia, Crawley, Australia; 3 Victoria University of Wellington, Wellington, New Zealand

**Keywords:** Autism, Infancy, Parental early concerns, Early behavioral concerns

## Abstract

Past research has highlighted the importance of early identification of developmental differences to improve targeted access to early interventions or supports. As such, it is of particular importance in the context of children at elevated likelihood of autism (such as where an older sibling has a diagnosis of autism), to better understand when and which early concerns are important as predictors of which children will benefit from pre-diagnostic supports. This study explored the number and frequency of retrospective parent reported concerns within the first year of life for children diagnosed with autism, both those who had an older sibling diagnosed with autism and those who did not, as well as for undiagnosed siblings. We found that at both 0–6 and 7–12 months, the only factor related to the presence or absence of early parent reported concerns was child diagnostic status, with the presence of reported early concerns more likely for children with a diagnosis of autism. These findings suggest that for children at elevated likelihood of autism, parents’ concerns are driven primarily by developmental differences, with child’s birth order and sibling diagnostic status not impacting on parent early concerns.

A growing body of research has found that commencing specific supports for Autism Spectrum Conditions (hereafter autism) prior to two years of age is beneficial in supporting developmental outcomes (Clark et al., 2018; Green et al., 2017; Landa, 2018; MacDonald et al., 2014; Whitehouse et al., 2021). These findings highlight the importance of early identification of developmental differences to improve targeted access to early interventions and supports (hereafter, ‘supports’) for those children who may benefit from them. While there is now increasing evidence that behavioural signs of autism (including reduced social attention and repetitive behaviours) are emergent over the first two years of life (for reviews, see Canu et al., 2021; Zwaigenbaum et al., 2015), the mean age of autism diagnosis globally is estimated to be around 5 years (van ’t Hof et al., 2021).

## Early Identification of Developmental Concerns

Early identification of autism generally starts with parents expressing developmental concerns about their child (e.g., De Giacomo & Fombonne, 1998). Families frequently express early concerns, with children who are developing differently often being referred to health professionals by their parents prior to 2 years of age due to concerns around their development (De Giacomo & Fombonne, 1998; Gibbs et al., 2019; Young et al., 2003). Parent reported concerns from 12 months have been shown to be associated with later behaviours associated with autism, and with later autism diagnosis (Hess & Landa, 2012; Ozonoff et al., 2009). Common concerns reported by parents include communication behaviours (such as delays in language acquisition, rare use of voice), delays in milestones (including delays in motor skills), and atypical social development and play (such as lack of eye contact, limited shared enjoyment or attention to caregiver, playing with toys in unusual ways) (Young et al., 2003).

## Evidence of Greater Developmental Concerns in Elevated Likelihood Versus Typical Likelihood Families

For siblings of autistic children, around 20% of younger siblings are later diagnosed with autism (Ozonoff et al., 2011), and a further 20–30% develop broader developmental difficulties (Messinger et al., 2013). As such, it is of particular importance in the context of elevated likelihood families (where an older sibling has a diagnosis of autism), to better understand when and which early concerns are important in determining later diagnostic status, and which children will benefit from pre-diagnostic support. There is evidence suggesting that parents of children with a diagnosis of autism do report a significantly greater number of early concerns for subsequent children when compared to parents of children without an autism diagnosis, regardless of diagnostic outcome of this younger child (Ozonoff et al., 2009; Sacrey et al., 2015; Talbott et al., 2015). While such studies have found that from 12 months and later there is an association between these concerns and later child diagnosis, elevations in reporting of early concerns occur before 12 months, sometimes unrelated to later child outcomes. For example, in a prospective study by Talbott et al. (2015) parents reported more early concerns if their child had an older sibling with autism compared to if they did not, but within this “elevated likelihood” group, there was no difference in the number of reported concerns for children who were subsequently diagnosed with autism and those who were not. Sacrey et al. (2015) found that overall, an elevated likelihood group later diagnosed with autism had more concerns reported by parents than did an elevated likelihood group not later diagnosed with autism, but that this group in turn elicited more concerns than a typical likelihood comparison group not later diagnosed with autism. One study also found that for children later diagnosed with autism, the presence of an older sibling with autism was associated with earlier reported parental concerns for subsequent children, even when developmental delays were less pronounced than when an older sibling with autism was not present (Herlihy et al., 2015).

Previous findings therefore indicate higher rates of parental reporting of early concerns when families already have a child diagnosed with autism, regardless of subsequent children’s later diagnostic status. These elevated concerns may reflect that parents may be hypervigilant or sensitised to detecting developmental differences in their younger child (Ozonoff et al., 2009), which could be exacerbated by their elevated stress, anxiety or depression (e.g., see Talbott et al., 2015). Alternatively, this could indicate, as has been suggested (Sacrey et al., 2015) that parents are identifying broader developmental and behavioural differences related to autism amongst the elevated likelihood subgroup who are not diagnosed with autism at study endpoint. As mentioned earlier, these broader differences are known to be elevated in this population (Messinger et al., 2013).

## Parental Sensitivity or Developmental Difference?

To examine whether parent reported early concerns are driven by increased parental sensitivity or are prompted by their child’s early developmental differences, the current study extended previous research by using a large community sample which provided access to additional subgroups typically not available in studies of elevated likelihood and typical likelihood siblings. The community sample included children diagnosed with autism, and any younger or older siblings, either diagnosed with autism or not. Table 1 presents the different comparison subgroups possible for examining the impacts of older sibling diagnostic status and the child’s own diagnostic status on parental early reporting. Where elevated likelihood samples compared to typical likelihood comparison groups have previously allowed for the comparison of subgroups 1, 2 and 3 (and possibly 6), our paradigm allowed the addition of subgroups 4, 5 and 6. These extra subgroups provide critical additional comparisons in investigating the impact of having an older autistic child on parental early concerns for a younger child. For instance, whereas previously this impact has been examined for younger children without an autism diagnosis (i.e., by comparing subgroups 2 and 3), our paradigm provides for the impact to also be assessed for younger children with an autism diagnosis (i.e., by comparing subgroups 1 and 4).

**Table 1:** Comparison subgroups as a function of older sibling diagnostic status and younger child’s own diagnostic status

Subgroup	Older Sibling Diagnostic Status	Child Diagnostic Status
1	Autism	Autism
2	Autism	No diagnosis
3	No diagnosis	No diagnosis
4	No diagnosis	Autism
5	No older sibling	Autism
6	No older sibling	No diagnosis

By including children diagnosed with autism and their diagnosed and un-diagnosed siblings, both older and younger, we aimed to examine the role of later diagnosis, as well as potential parental sensitisation due to an older child with autism on the reporting of early concerns. We hypothesised firstly, that younger siblings of a child diagnosed with autism (subgroups 1 and 2) would have increased parental reporting of early developmental concerns when compared to children who did not have an older sibling with an autism diagnosis (subgroups 3, 4, 5 and 6). Secondly, we hypothesised that this difference would be observed for both the younger siblings who receive an autism diagnosis and those who do not. We also expected more reports of early concerns for the younger siblings who later received an autism diagnosis compared to those who did not.

## Methods

### Participants

This study used data collected as part of the Australian Autism Biobank (AAB), a biobank that includes phenotypic data from children with a diagnosis of autism (confirmed by formal diagnostic reports provided by parents, and assessment of children through the Autism Diagnostic Observation Schedule-2 [ADOS-2; Lord et al., 2012] by researchers), aged between 2 and 17 years, as well as their parents and siblings. For more information on the recruitment, sample and data collection of the AAB sample, please refer to Alvares et al. (2018). For the current study a waiver of consent was also approved by the University of Western Australia, for data and samples previously collected (2021/ET000928). Additionally, access to the data was approved by the Autism CRC Access Committee. For the current study, we focussed on a subgroup of 525 children from this biobank (male: *n* = 399; female: *n* = 126), 438 of whom were probands (male: *n* = 353 male; female: *n* = 85), and 87 siblings (male: *n* = 46; female: *n* = 41). Participants were included only if they were under the age of 6 years at the time of participation (therefore aged between 2 and 6). We focussed on this subgroup to increase the chance that parents would be able to accurately recall concerns around child development in the first year of life, following a similar rationale to previous studies (Sainsbury et al., 2022; Waddington et al., 2022). Included in the study were participants (1) with a diagnosis of autism, and (2) undiagnosed children with a sibling with autism. Data were included from participants who were under the age of 6 and whose caregivers had completed the section of a Family History Questionnaire (FHQ) created for the AAB containing questions on early development including early concerns.

### Measures

#### Family History Questionnaire (FHQ)

The report of early parental concerns was drawn from the FHQ. This questionnaire was developed specifically for use for the AAB (Alvares et al., 2018). The FHQ contains 8 subsections including family structure and demographic information, biological maternal and paternal health and medical history. It also covers the pregnancy, birth, development, and medical history for each child in the family. For the current study, only demographic information and child development information were used. This information was collected for probands (children diagnosed with autism who were the primary subject of this study), and for siblings, with family structure information used to calculate child parity. There was also a record within the biobank database as to whether or not each child had a sibling(s) with a diagnosis of autism, and the age of that sibling, which was used to group probands as being from simplex (families with only one child with an autism diagnosis), or multiplex families (where more than one child had an autism diagnosis), and to calculate whether each proband from a multiplex family was the eldest, or subsequent child in the family, with a diagnosis of autism. The child development section in the FHQ contained two questions relevant to early developmental concerns. Parents were advised to refer to their child’s “baby book” (health records of child health nurse visits, immunisations, and physical measurements, amongst other health information), if available, for help in answering: “*Do you recall anything unusual about your child’s development or behaviour during their first 6 months?”* and *“Now, think of the period between 6 and 12 months, can you recall anything unusual about your child’s development or behaviour during that time?”* Parents could respond yes or no to these two questions, and if they responded yes, there was an open text box where they could provide more details about the specific aspects of atypical development that they noticed for each age.

### Data Coding

The parent free-text responses detailing early concerns at 0–6 months and 7–12 months were coded for probands in a recent study (for a full description, see Waddington et al., 2022), based on the domains and corresponding subdomains outlined by previous research (Guinchat et al., 2012). The domains relate to: (a) language, (b) social development, (c) stereotyped/restricted behaviour, (d) motor development, (e) behaviour/temperament, (f) medical issues, (g) abnormal physiological function (i.e., difficulties with sleeping and feeding), (h) other idiosyncratic development, (i) atypical physical features and (j) unspecified autism concerns.

For the current study, the same coding protocol (Waddington et al., 2022) was implemented here to code parent responses to the same questions for sibling data from the Australian Autism Biobank, to explore these responses in comparison with the existing proband codes. This involved the coding of the presence of concerns in each domain as listed above, and for up to 9 corresponding subdomains; for example, the subdomains for language included: (a) delayed speech/vocalizations, (b) no speech/vocalizations, (c) poor language comprehension, (d) language regression, (e) lack of language imitation, and (f) other atypical language. A score of 1 in each domain/subdomain indicated that the aspect of atypical development was present in the parent response, while a score of 0 indicated that it was not. All responses were coded by one author (DC), with a second author who has previous experience with the coding protocol (EM) coding 20% of responses at each age. Item level coding agreement was calculated using the formula agreements/(agreements + disagreements) × 100, with sub-domain-level agreement at 100% at ≤ 6 months, and 86% at 7–12 months. All disagreements were resolved by consensus between the two authors.

### Data Analyses

Data were collated in Microsoft Excel and exported to IBM SPSS statistics v28, through which all analyses were conducted. Descriptive statistics were used to assess demographic characteristics of families, multiplex or simplex family status and the frequency of parental concerns across each group. To examine the relationship between the presence or absence of early developmental concerns and the presence of an older sibling or the presence of an older autistic sibling, children’s dates of birth, diagnostic status, and family ID were used to compute variables indicating whether each child had an older sibling, and whether this older sibling was diagnosed with autism. Siblings included in these analyses were genetic full-siblings and maternal half-siblings, to most accurately capture the parental experience of having previously parented an infant, as the majority of family history questionnaires were completed by mothers (72.6%) or by both biological parents together (19.4%). Twins were considered equal in birth order (e.g., if a child had a twin sibling and the family had no prior children, they were both considered first in parity).

To examine whether the presence or absence of early concerns was associated with the presence or absence of older siblings or whether this older sibling was autistic, a multi-level binary logistic regression was used at both time periods (0–6 and 7–12 months). Variables included in the analyses were firstly, a variable of older sibling status coded to indicate (1) the presence of an older sibling diagnosed with autism, (2) the presence of an older sibling without an autism diagnosis, and (3) no older sibling. Category 1-the presence of an older sibling diagnosed with autism, was used as the reference category in analyses. Secondly a variable indicating the participant’s own autism diagnostic status as (1) diagnosed, or (2) not diagnosed was included. These variables were used to examine the effects of older sibling status and participant diagnostic status on the outcome variable of the (1) presence or (2) absence of parent reported early concerns. The participant’s sex was also included in the model, and to account for the clustering effect of families, Family ID was used as a random effect in the model.

To further examine whether there were any differences in the number of domains of early concerns reported by families at 0–6 months and at 7–12 months associated with older sibling status and participant status, for each time point a multi-level loglinear regression with a Poisson distribution was conducted using the same predictor variables described above, and with the number of domains reported as the outcome variable. A robust estimation to handle violation of model assumptions was used to address the zero inflation that was present in the data. The datasets supporting the conclusions of this article are available by application to the Australian Autism Biobank within the Cooperative Research Centre for Living with Autism (Autism CRC): https://www.autismcrc.com.au/biobank.

## Results

The final sample included 525 unique individuals (male: *n* = 399; female: *n* = 126), 438 of whom were probands (male: *n* = 353 male; female: *n* = 85), and 87 siblings (male: *n* = 46; female: *n* = 41). Of the probands, 101 belonged to a multiplex family, where more than one child was diagnosed with autism. Probands’ mean age was 51 months and their mean age when they received their autism diagnosis was 39 months. Mean age of siblings was 47 months. The parents who completed the FHQ were most commonly Caucasian, had completed university, and earned more than AUD$104,000 per year in household family income. More detailed demographics of the sample are reported in Alvares et al. (2018).

Table 2 shows the results of the multi-level binary logistic regression examining the effects of sibling status (no older sibling, the presence of an older sibling without an autism diagnosis, or the presence of an older sibling diagnosed with autism), participant own diagnostic status, and child sex on presence of early concerns. At both 0–6 and 7–12 months, the only factor related to the presence or absence of parent early reported concerns was child diagnostic status, with the presence of reported early concerns more likely for children with a diagnosis of autism. At 0–6 months early concerns were reported for 44% of children later diagnosed with autism, and for 9% of children not later diagnosed. At 7–12 months early concerns were reported for 62% of children later diagnosed with autism, and 18% for those who were not. Surprisingly, sibling status did not impact on the presence of early concerns, with concerns no more frequent for children with an older sibling diagnosed with autism compared to either children who had an older non-autistic sibling or children who had no older sibling at all (see Table 2).

**Table 2:** Association between parent-reported early concerns and older sibling status, participant diagnostic status and sex for 0-6 month age group, and for 7-12 month age group

	Comparison	B (SE)	*p*	OR (95% CI)
**0–6 months**				
Older sibling status				
1. Diagnosed with autism				
2. Not diagnosed with autism	1 vs. 2	-0.23 (0.29)	0.42	0.79 (0.45, 1.39)
3. No older sibling	1 vs. 3	0.05 (0.26)	0.84	1.05 (0.63, 1.77)
Diagnostic status	No ASD vs. ASD	2.04 (0.42)	**< 0.001**	7.69 (3.35, 17.64)
Child sex	M vs. F	-0.12 (0.23)	0.62	0.89 (0.56, 1.41)
**7–12 months**				
Older sibling status				
1. Diagnosed with autism				
2. Not diagnosed with autism	1 vs. 2	-0.06 (0.30)	0.84	0.94 (0.53, 1.68)
3. No older sibling	1 vs. 3	0.19 (0.27)	0.50	1.21 (0.70, 2.07)
Diagnostic status	No ASD vs. ASD	1.92 (0.36)	**< 0.001**	6.82 (3.37, 13.82)
Child sex	M vs. F	-0.10 (0.24)	0.68	0.91 (0.57, 1.44)

*Note*: In each comparison the reference level is listed first. Exclusions due to missing data, *n* = 6

Table 3 shows the results of the multi-level loglinear regressions examining the number of coded domains endorsed by parent early concerns. Again, the only factor related to the number of domains reported by parents was child diagnostic status, and only for the time period of 7–12 months. For this period, the mean number of domains reported were 1.29 (SD = 1.39) for children later diagnosed, and 0.26 (SD = 0.77) for children not later diagnosed with autism.

**Table 3:** Association between number of domains of parent-reported early concerns and older sibling status, participant diagnostic status and sex for 0-6 month age group, and for 7-12 month age group

	Comparison	B (SE)	*p*	OR (95% CI)
**0–6 months**				
Older sibling status				
1. Diagnosed with autism				
2. Not diagnosed with autism	1 vs. 2	0.03 (0.14)	0.84	1.03 (0.79, 1.34)
3. No older sibling	1 vs. 3	0.22 (0.12)	0.07	1.25 (0.98, 1.59)
Diagnostic status	No ASD vs. ASD	0.29 (0.15)	0.06	1.34 (0.99, 1.81)
Child sex	M vs. F	-0.07 (0.10)	0.47	0.93 (0.76, 1.14)
**7–12 months**				
Older sibling status				
1. Diagnosed with autism				
2. Not diagnosed with autism	1 vs. 2	0.03 (0.16)	0.88	1.03 (0.75, 1.40)
3. No older sibling	1 vs. 3	0.02 (0.15)	0.88	1.02 (0.77, 1.36)
Diagnostic status	No ASD vs. ASD	1.59 (0.25)	**< 0.001**	4.90 (3.01, 7.96)
Child sex	M vs. F	-0.01 (0.13)	0.97	1.00 (0.78, 1.28)

*Note*: In each comparison the reference level is listed first. Exclusions due to missing data, *n* = 29

## Discussion

The current study explored parent-reported early concerns to better understand how the nature and timing of early concerns might relate to the presence or absence of an older sibling with or without a diagnosis of autism, in a sample where all children would be considered at elevated likelihood of autism. We compared the reported early concerns for children with and without a diagnosis of autism, to examine the impact of parity as well as diagnostic status. Our results found no difference in the retrospective reporting of early concerns related to the presence of an older sibling, or their autistic status. Furthermore, our results showed that the only significant predictor of the presence or absence of parental early concerns was child’s later diagnostic status. Thus, contrary to one of our hypotheses, younger siblings of a child diagnosed with autism (subgroups 1 or 2 as introduced in the introduction) did not have increased parental reporting of early developmental concerns when compared either to children with an older non-autistic sibling or to children with no older siblings (subgroups 3–6).

These findings differ from previous research that has examined reported concerns both retrospectively and prospectively for infant siblings of children diagnosed with autism, where these infants are followed up to establish diagnostic outcome. Such studies found that parents reported more early concerns if their child had an older sibling with autism compared to if their older sibling did not have a diagnosis of autism (Ozonoff et al., 2009; Talbott et al., 2015); in contrast, the results of the current study showed that only child diagnostic status was associated with early parental concerns.

The discrepancy between our findings and those of previous studies could indicate that previous findings of elevated parental concern where an older child has autism might be due to increased incidence of children from elevated likelihood families going on to have later developmental difficulties as well as increased autism diagnoses; with this not captured where comparison is typically based on autism diagnostic outcome at 24–36 months. It has also previously been acknowledged that this elevation could in part reflect a selection bias of recruitment to elevated likelihood samples, where parents who already have concerns about development are more likely to enrol in a study (Ozonoff et al., 2009). Through the implementation of a large sample of community diagnosed children and their siblings, the present study may have minimised this selection bias.

Through the methodological advantages of the current study in access to a large community diagnosed sample, and inclusion of data on early concerns and diagnostic status for all children in the family enabling inclusion of extra comparisons of children than previous research, the current study has allowed for the separation of the influence of the child’s diagnostic status from the influence of older sibling status on parent concerns. While it has been previously suggested that in having an older autistic child, parents may be hypervigilant or sensitised to the reporting of early concerns, our results would suggest that this is not the case, with results suggesting that parent concerns are driven primarily by developmental differences, with child birth-order and sibling diagnostic status not impacting on parent early concerns.

A limitation of the study design is that data were based on a single questionnaire and collected retrospectively through parental recall, rather than through the ideal of prospective assessment. It is possible that the time elapsed between early childhood behaviour and parental recall may have influenced the quantity and quality of concerns recalled. We attempted to minimise the impact of this through limiting the sample to children aged under 6, such that the early developmental period was more likely to be recalled more accurately, and the AAB attempted to improve accuracy in answering by prompting parents to use child development records to assist them in answering the questionnaire. However, findings should be interpreted in light of this limitation, and the need for replication. Prospective collection of data of this nature raises logistical challenges with regard to requiring a large sample, especially for recruiting sufficient families with children without a diagnosis of autism, where future children will go on to be diagnosed with autism. However, future research examining parental early concerns through such an approach would help to further establish the potential role of early reported concerns, and their association to later autism diagnosis. Of particular interest would be to further determine how parental early concerns relate to broader factors such as parent-infant interactions (as a known focus of early supports). Also warranting further investigation is the direct examination of parental traits of anxiety or hypervigilance on early reported concerns, as well as the influence of other factors not explored in the current study, such as parental awareness of autism and knowledge of early development.
